# Understanding motivation for implementing cooperative learning methods: a value-based approach

**DOI:** 10.1007/s11218-021-09666-3

**Published:** 2021-12-10

**Authors:** Dimitra Filippou, Céline Buchs, Alain Quiamzade, Caroline Pulfrey

**Affiliations:** 1 Faculty of Psychology and Educational Sciences, University of Geneva, Boulevard Du Pont d’Arve 40, 1211 Geneva, Switzerland; 2 Faculty of Social and Political Sciences, University of Lausanne, Lausanne, Switzerland

**Keywords:** Cooperative learning, Personal values, Contextual values, Teachers’ values

## Abstract

The implementation of cooperative learning methods remains disparate in primary schools despite their widely recognised benefits. To explain this paradox, we first examined whether teachers’ inclination towards cooperative methods is motivated by their values. Second, we tested whether motivational connections between personal values and cooperative methods are undermined when conflictual values are activated in context. Study 1 demonstrated that pre-service teachers strongly endorsed self-transcendence (ST) values (expressing compatible motivations with cooperation) relative to self-enhancement (SE) values (expressing conflictual motivations with cooperation). Adherence to ST values was also positively associated with their beliefs and attitudes regarding cooperative methods. In Studies 2, 3 and 4, educational sciences students were experimentally exposed to different contexts, wherein ST, SE or neutral values were promoted. Our findings indicate that when SE values were emphasised in the context, the positive association between ST values and beliefs/attitudes regarding cooperative methods disappeared. Although the results of Study 4 regarding the intention to use cooperative methods were not statistically significant, the pattern was similar. Finally, Study 5 showed that primary school teachers’ ST values positively predicted the self-reported use of cooperative methods when they perceived their school to weakly endorse SE values, but not when they perceived it to strongly endorse them.

## Introduction

In recent decades, the effectiveness of cooperative learning methods has been widely recognised in the research community. Based on robust empirical results, many scholars have been strongly encouraging their use in classrooms, especially in primary schools (Gillies, 2016; Johnson & Johnson, 2002; Slavin, 2015). Cooperative learning methods may refer to any form of learning strategy in which learners actively work together towards joint goals to achieve academic outcomes for not only themselves but also other group members (Johnson & Johnson, 1999; Slavin, 2014). Often compared with individualistic or competitive methods, cooperative methods have been consistently demonstrated to be more beneficial for academic achievement (Balta et al., 2017; Kyndt et al., 2013; Roseth et al., 2008), well-being (Hanson et al., 2016; Van Ryzin & Roseth, 2018) and social relationships (Roseth et al., 2008; Tolmie et al., 2010). Not surprisingly, cooperative methods are not only recommended by educational researchers, but professionals in the educational field also seem to acknowledge their positive influence on pupils’ social and learning outcomes (Ruys, van Keer, et al., 2010; Tal, 2018).

Despite their reported benefits, several studies have shown that the application of cooperative methods remains marginal in classrooms (Baines et al., 2003; Buchs et al., 2017; Pianta et al., 2007; Wasik, 2008). To extend our understanding of cooperative instruction, we examined a hypothesis that incorporates teacher-related variables at personal and contextual levels. More precisely, we investigated whether the interplay between personal and contextual values affects individuals’ motivation to implement cooperative learning methods.

We first examined the association between personal values and cooperative learning inclination. Previous research has shown that individuals choosing a career in teaching are likely to endorse values expressing compatible motivational connections with cooperation (Ros et al., 1999; Vansteenkiste et al., 2006). Hence, teachers might be inherently motivated—guided by their values—to promote cooperative methods. However, the translation of personal values into actions is often subject to the influence of contextual values (Roccas & Sagiv, 2010). In the context of education, normative grading practices, excellence strivings, high-stakes testing and certification standards emphasise *self-enhancement* (SE) values of power and achievement (Branco, 2018; Connell, 2013; Kumar & Maehr, 2007; Reeve & Assor, 2011; Veugelers & Vedder, 2003) and may hinder teachers’ value-based motivation to implement cooperative methods. Thus, conflicting values may operate as countervailing forces that hinder individuals’ value-expressive practices. We argue that the discord between individual and contextual values may explain the weak place of cooperative methods in classrooms.

### Cooperative learning methods and teachers’ role in their implementation

Cooperative instruction involves learners actively working together, in small groups, towards common goals. It is based on the principle of positive interdependence, which posits that one’s actions positively influence those of others (Butera & Buchs, 2019; Johnson & Johnson, 1989, 2002). In cooperative settings, learners actively participate in knowledge construction by interacting with other group members. For instance, to attain the group’s common goal, group members are encouraged to help each other, discuss their views and exchange information. These constructive interactions (Slavin, 1996, 2014) along with an emphasis on cohesiveness (Battistich et al., 1993) and prosocial goals, which include mutual help, empathy and reciprocity (Levontin & Bardi, 2018), contribute to enhancing learning outcomes.

Teachers play an important role in ensuring the effectiveness of these methods (Gillies, 2016). They are responsible for proposing adequate activities, preparing pupils for cooperation and structuring cooperative work around a shared goal (Topping et al., 2017). Empirical findings suggest that introducing cooperative work in the classroom is often perceived as challenging by teachers, as this pedagogical method requires adequate training and knowledge (Gillies & Boyle, 2010; Koutselini, 2009). Therefore, teachers’ professional competence plays a prominent role in the application of cooperative methods. Yet, it has been argued that, alongside their competence teachers may also require motivation, devotion and intentional efforts, to ensure sustainable use of cooperative pedagogy (Tal, 2018). Such a perspective seems to be in line with a body of research that emphasises the relevance of addressing teachers’ inherent motivations and needs, to better understand their instructional orientations, professional engagement and teaching effectiveness (Barni et al., 2019; Han & Yin, 2016; Liou et al., 2019; Reeve, 2009). A way to investigate inherent motivations is to examine individuals’ values (Barni et al., 2018, 2019; Levontin & Bardi, 2019). Therefore, in this research, we aimed to understand teachers’ motivations to apply cooperative methods based on personal values.

### The role of values in explaining beliefs, attitudes and behaviours

Values constitute a central construct characterising a culture (Schwartz, 1996, 2006). Within a social system, values are manifested at two levels (Schwartz, 2014): societal/contextual and individual. At the individual level, values refer to abstract, desirable goals that transcend specific situations and serve as guiding principles in a person’s life (Schwartz, 1992, 2017; Schwartz et al., 2012). At the contextual level, values denote what is important within a specific context and may have a *prescriptive role* when individuals perceive that they are expected to comply with these values (Schwartz, 2014; Vauclair et al., 2015).

Schwartz (1992) identified 10 universal values that are placed around a circular continuum and can be summarised in four high-order categories of values, which form two opposing dimensions. The first dimension contrasts *openness to change* (stimulation, self-direction and hedonism) with *conservation* values (security, tradition and conformity). The second dimension, which is more relevant to the current study, contrasts *self-transcendence* (ST) values with self-enhancement (SE) values. ST values include universalism values, which express motivational goals for the understanding and tolerance of all people and nature, and benevolence values, which emphasise the pursuit of welfare protection of those around us. In contrast, SE values encompass power values, which express motivations for social status, prestige and dominance over others and achievement values, which express motivations for personal success and demonstration of competence according to social standards. One of the most important features of values is that they represent a system of basic motivations and drive people to work towards them (Schwartz, 2011). Therefore, the more one considers a value important, the greater the motivation to attain goals that reflect this value. Numerous studies have established the predicting role of personal values on motivationally compatible beliefs^1^ (de Groot & Steg, 2008; Rohan, 2000; Rokeach, 1968; Stern et al., 1995), attitudes (Boer & Fischer, 2013; Maio & Olson, 1994) and behaviours (Lönnqvist et al., 2013; Maio et al., 2009; Schwartz et al., 2012; Skimina et al., 2019; Verplanken & Holland, 2002). Interestingly, another study revealed that values not only are positively associated with behaviours that express a value but also can inhibit behaviours related to motivational goals, inconsistent with this value (Feiler et al., 2012).

Schwartz’s theoretical model further proposes that a person's values vary in importance, and it is this relative adherence (i.e., the importance attributed to one type of value relative to all other values) that guides individuals’ lives. Thus, since values operate as an integrated system, one's adherence to each value must be estimated by considering the position of that value relative to the complete set of values (Bilsky et al., 2015; Schwartz et al., 2012).

### Teachers’ values and cooperative learning methods

Considering that personal values can guide individuals’ lives, we assumed that they would also be reflected in their career choices. Studies have shown that teachers and pre-service teachers are likely to prioritise ST values over other types of values (Ros et al., 1999; Vansteenkiste et al., 2006). The strong endorsement of ST values among those choosing this career path is quite conceivable if we consider that the profession of teaching involves caring about pupils’ learning and social outcomes (Wentzel, 2004). Thus, we expected that individuals choosing a teaching career endorse ST values more than SE values.

More importantly, given the apparent determinant role of values, they may also influence teachers’ instructional learning choices. Teachers’ values predict their instructional orientations and teaching styles (Barni et al., 2018, 2019; Cai et al., 2002; Hadar & Benish-Weisman, 2019). Therefore, this may also explain their motivation towards cooperative learning methods. Findings from studies from different domains suggest that cooperative and competitive behaviours (Hinz et al., 2005; Sagiv et al., 2011; Schwartz, 1996) or goals (Levontin & Bardi, 2019) are associated with the value dimension of ST—SE values. Specifically, this body of research suggests that ST value endorsement is positively associated with cooperative behaviours and negatively with competitive behaviours. The inverse pattern of associations holds for SE values.

By analogy, we assumed that cooperative learning situations—in which learners are expected to share their knowledge, provide help and respect each other—underlie compatible motivations with ST values and incompatible motivations with SE values. In contrast, competitive learning situations—in which pupils seek to outperform their peers to attain a reward that only one or a few can achieve (Elliot et al., 2016; Johnson & Johnson, 1975, 1999)—underlie compatible motivations with SE values and incompatible motivations with ST values. Therefore, we suggest that the endorsement of ST values will orient teaching behaviour in favour of cooperative methods.

### The interplay between personal and contextual values

Several studies have underscored the importance of considering contextual characteristics when we investigating value expression attitudes and behaviours (Boer & Fischer, 2013; Pulfrey et al., 2017; Roccas & Sagiv, 2010; Rudnev & Vauclair, 2018). Overall, these findings indicate that the strength of the relationship between personal values and actions or attitudes may vary across contexts with different prevailing values (Boer & Fischer, 2013; Rudnev & Vauclair, 2018), or it may be reduced when normative pressures—conveying opposing views to one’s values—are present (Bardi & Schwartz, 2003).

Interestingly, previous studies have provided some evidence for the significant role of context in the implementation of cooperative learning methods. First, at a macro-cultural level, two recent meta-analyses (Balta et al., 2017; Kyndt et al., 2013) suggested that the effectiveness of cooperative methods on academic achievement, as compared with other methods (competitive and individualistic learning), was weaker in Western cultures, where self-interest values are dominant. Second, and more proximal to the school level, Pulfrey and Butera (2016) indicated that contextual values moderate the association between personal values and behaviour among university students. Specifically, they observed that the negative association between ST values and academic cheating was undermined when SE values were made experimentally salient in the context. This appears consistent with the argument that the presence of values in a context that conflicts with an individual’s values reduces the likelihood of value expression behaviours in such a situation. Third, concerning teachers themselves in the classroom, Assor (1997) reported that teachers’ endorsement of the value of independence in education predicted value-consistent teaching behaviours in the classroom, but only when this value was chronically accessible in their context. Consistent with this view, another study using a qualitative methodology (Hornstra et al., 2015) found that when teachers perceived contextual pressures, such as national performance standards, high-stakes testing and pressures of school administration, they reported teaching behaviours that did not converge with their personal teaching preferences. Overall, these findings stress the importance of studying the role of teachers by also considering the specific characteristics within their context.

For teachers, school constitutes a context that is likely to influence them and their teaching behaviours (Pelletier & Sharp, 2009; Reeve, 2009). Schools operate as societal institutions to fulfil different functions (Connell, 2013; Friedman, 2003; Reeve & Assor, 2011) that may express both ST and SE values. For instance, on the one hand, schools may entail missions that are compatible with ST values such as promoting prosocial behaviours, implementing inclusive and egalitarian policies and supporting equal opportunities for all pupils (Sahlberg, 2016). On the other hand, schools are often responsible for sorting pupils according to their performance, implementing normative evaluation and certification standards (Autin et al., 2015; Batruch et al., 2019; Sahlberg, 2016). Such practices emphasising motivations of self-interest, performance demonstration and social recognition would arguably render SE values particularly relevant in schools and could presumably affect cooperative learning use. Accordingly, experimental studies have found that the presence of normative grading practices (compatible with SE values) in learning situations hamper cooperation among pupils (Burleigh & Meegan, 2018; Hayek et al., 2017). Thus, we hypothesised that when contextual value priorities conflict with teachers’ value priorities, the former can hinder the expression of the latter. Particularly, we propose that when SE values are present in the context, they can reduce the positive association between ST personal values and readiness for cooperative learning methods.

### Overview of studies

Study 1 aimed to investigate motivational connections between personal values and cooperative methods. We tested the hypothesis that relative adherence to ST values at the individual level is positively associated with beliefs about and attitudes towards cooperative learning methods. It also aimed to demonstrate that ST values are highly endorsed by individuals choosing a career in teaching.

Afterwards, a series of studies were designed to examine the moderating role of contextual values on the link between individuals’ relative adherence to ST values and cooperative learning methods in terms of beliefs, attitudes and behavioural intentions.

In Studies 2, 3 and 4, after measuring the relative endorsement of ST values, we experimentally manipulated contextual values (ST values vs. SE values vs. control) at different levels—proximal or distal to the classroom—that were likely to affect individuals. More precisely, Study 2 tested whether contextual values at the societal level affected the link between relative adherence to ST values and a) beliefs and b) attitudes regarding cooperative learning methods among students of educational sciences. In Study 3, we used teaching material as a prime to experimentally manipulate contextual values and we tested their effect on the link between ST value adherence and beliefs about cooperative learning methods among students in educational sciences. Study 4 extended the results of the two previous studies by using an outcome variable closer to actual behaviour. More precisely, we measured behavioural intentions to use cooperative methods among pre-service teachers. Contextual values, in this study, were manipulated at the organisational level of the school.

Finally, Study 5 attempted to extend the results to real settings by focusing on the self-reported use of cooperative methods (i.e., real behaviour) among in-service schoolteachers. More precisely, it aimed to test the extent to which perceived SE values in the context (at the school level) moderate the link between teachers’ relative adherence to ST values and the frequency with which they use cooperative methods.^2^

All studies reported in this article were approved by the ethics committee (Commission Ethique-FPSE) of the Faculty of Education and Psychology at the University of Geneva and none of them were associated with any risk to participants. The confidentiality and data protection requirements comply with the guidelines of the ethics committee in force at the time of data collection. In addition to the approval of the ethics committee of the Faculty of Education and Psychology at the University of Geneva, Study 5 was also approved by the Geneva public education service as it was conducted among in-service teachers employed in Geneva public schools. Accordingly, in all studies reported in this paper, before giving their consent, respondents were informed about the purpose and implications of the research, along with the data confidentiality and protection regulations. In all cases, participation was voluntary and thus participants were aware of their right to refuse to participate or to withdraw their participation at any time.

## Study 1

In Study 1, in accordance with our first hypothesis, we examined whether pre-service teachers prioritise ST over SE values. We then tested whether relative adherence to ST values was associated with pre-service teachers' beliefs and attitudes regarding cooperative and competitive learning methods. Considering that cooperation and competition are both defined by interdependence between learners (Deutsch, 1949), in this study, we introduced both cooperative and competitive methods toverify that ST values were positively linked with cooperation and negatively linked with competition. We further aimed to confirm that the hypothesised positive association with ST values is specifically attributed to cooperative methods and not to other instructional methods.

From the perspective of social interdependence theory (Deutsch, 1949, 1962), competition is conceptualised as the opposite of cooperation. Unlike cooperation, which is built on a positive interdependence between members, competition is built on a negative interdependence between members. In other words, in a competitive situation, a win for an individual implies a loss for others. Thus, when pupils learn in competition they aim to achieve a goal or rewards that only one or few pupils can attain (Johnson & Johnson, 1989).

Based on this theoretical conceptualisation, and on results from previous studies on associations between ST values and cooperation and competition (e.g., Sagiv et al., 2011), we formed the following bidirectional hypothesis: individuals’ relative adherence to ST values will be related a) positively to attitudes towards, and beliefs about, cooperative learning methods (expressing compatible motivations); and b) negatively to attitudes towards, and beliefs about, competitive learning methods (expressing incompatible motivations). Existing studies indicate that teaching beliefs and attitudes may influence teaching behaviours and are therefore crucial components in understanding instructional practices (Hermans et al., 2008; Lumpe et al., 1998). Research has further underscored the relevance of studying beliefs and attitudes among future teaching professionals, as they are especially important from the early stages of their career (Richardson, 1996, 2003).

### Method

#### Participants/procedure

Two hundred and thirty pre-service primary schoolteachers of a French-speaking Swiss university voluntarily accepted to participate in the study.^3^ Participants were in the 3rd year of their four-year training and recruited over the duration of four years^4^ either during a university course (paper and pencil format) or over the Internet. They were informed that the aim of the study was to find out about their views on different instructional methods implemented in classrooms in primary school and were asked to complete a questionnaire. Participation was anonymous. Demographic data revealed 84.04% of the respondents were women and the average age of the total sample was 24.42 years (*SD* = 4.76).

#### Measures

##### Personal values

Participants’ values were measured with an adapted, shortened version^5^ (Pulfrey & Butera, 2013, 2016) of Schwartz’s Portrait Value Questionnaire (PVQ; Schwartz et al., 2001). The shortened version was preferred because of time restrictions. Participants were asked to report, on a 7-point scale (1 “not at all important” to 7 “absolutely important”), how important the different statements pertaining to one of the four high-order categories of values were to them: ST (8 items, e.g., “It is important to me to be that every person in the world is treated equally”, α = .79), SE (7 items, e.g., “It is important to me to be successful”, α = .82), openness to change (9 items, e.g., “It is important to me to think up new ideas and to be creative”, α = .73), and conservation (10 items, e.g., “It is important to me to follow the rules and do what one is told”, α = .69).

In Schwartz’s (2006) methodology, values are ordered according to their importance; the relative importance between each value guides individuals’ lives. Thus, according to Schwartz, it is mandatory to compute relative (centred) scores for values. To calculate each relative value, one should first average the individual ratings for all items corresponding to each type of value. Then, from each value type mean score, the individual’s overall mean across all value items has to be subtracted (e.g., ST values = raw mean of ST values—individual mean score for all scale items). Partialling out an individual’s mean for all value items has been shown to provide stronger and theoretically more meaningful relationships, compared to the use of raw values scores (Borg & Bardi, 2016; Parks-Leduc et al., 2015). It may also consist of an efficient way to account for individual differences in scale use (e.g., some respondents tend to agree with all items (acquiescence bias), Winkler et al., 1982). Therefore, relative adherence to ST values used throughout this paper refers to the importance attached to ST after subtracting the mean ratings for all values items (see also Bilsky et al., 2015).

##### Beliefs and attitudes regarding cooperative and competitive learning methods

To measure beliefs and attitudes about cooperative and competitive learning methods, we developed a range of items based on an existing scale (Johnson & Norem-Hebeisen, 1979). Participants indicated their agreement on a 7-point Likert scale (1 “not at all agree” to 7 “completely agree”). A confirmatory factor analysis with a Satorra-Bentler scaled chi-square test and robust standard errors (Satorra & Bentler, 1994; Savalei, 2014) was conducted on the four latent variables (belief vs. attitude measures about cooperative vs. competitive learning). The four-variable model showed satisfactory goodness of fit indices (χ^2^_Satorra-Bentler_ = 80.80, *df* = 47, *p* = .002, RMSEA _Satorra-Bentler_ = 0.057, SMREA = 0.044, CFI_Satorra-Bentler_ = 0.97). Items with their standardised factor loadings are presented in Table 1 along with the descriptive statistics of each variable.

**Table 1:** Confirmatory factor analysis

Factors	Scale and items	Factor loading
Beliefs about “competitive” learning methods (α = .86) (*M* = 2.35, *SD* = 1.12)	Competition amongst pupils motivates them to work	.80
	Encouraging pupils to be better than others stimulates learning	.80
	Competition amongst pupils is a good way for them to work and learn	.86
Beliefs about “cooperative” learning methods (α = .70) (*M* = 6.03, *SD* = .80)	Mutual aid amongst pupils is a good means to work and learn	.75
	Cooperation amongst pupils motivates them to work better	.58
	Pupils learn a lot of important things from each other when they share their ideas and the means they have at their disposal	.63
Attitudes towards “competitive” learning methods (α = .82) (*M* = 1.88,*SD* = 1.06)	I wish that my pupils would work for better grades than the others	.64
	I wish that my pupils would seek to do better work than the others	.91
	I wish that my pupils would seek to be the best of the classroom	.81
Attitudes towards “cooperative” learning methods (α = .78) (*M* = 6.42, *SD* = .71)	I wish that my pupils would share their ideas and the means they dispose	.85
	I wish that my pupils would cooperate	.69
	I wish that my pupils would help each other during their learning process	.71

Standardised factor loadings for items measuring beliefs and attitudes regarding cooperative and competitive methods (Study 1)

All loadings were significant, *p* < .001

Testing the discriminant validity between beliefs and attitudes. Since correlations between beliefs and attitudes (about cooperative [*r* = 0.68] and competitive [*r* = 0.61] learning methods) were strong, we tested their discriminant validity using the model comparison approach (Zaiţ & Bertea, 2011). We compared the model of four factors against a two-factor model in which beliefs and attitudes were set to pertain to a common latent factor. The likelihood ratio test showed that the four-factor model was significantly better than the two-factor model (χ^2^ = 104.20, *df* = 2, *p* < .001). In other words, beliefs and attitudes were identified as discriminant constructs and therefore were treated as two different variables in the following analyses.

### Results

The results^6^ of the paired sample t-test indicated that participants endorsed ST values to a greater extent (*M* = 5.96, *SD* = 0.68) than they did the opposing SE values (*M* = 4.04, *SD* = 0.99), *b* = 1.93, *SD* = 0.08, *t*(228) = 25.06, *p* < .001, 95% CI [1.77, 2.08]. The difference between beliefs about cooperative methods (*M* = 6.03, *SD* = 0.80) and beliefs about competitive methods (*M* = 2.35, *SD* = 1.12) was also significant according to the paired sample t-test and weighted in favour of cooperative methods, *b* = 3.68, *SE* = 0.10, *t*(223) = 37.90, *p* < .001, 95% CI [3.49, 3.87]. Similarly, participants reported more positive attitudes towards cooperative methods (*M* = 6.42, *SD* = 0.71) than towards competitive methods (*M* = 1.88, *SD* = 1.06), *b* = 4.54, *SE* = 0.09, *t*(223) = 48.28, *p* < .001, 95% CI [4.36, 4.73].

To examine whether ST value adherence was associated with beliefs and attitudes, we used relative scores of ST values, as described in the method section. Spearman’s correlation coefficients revealed that participants’ relative adherence to ST values was positively linked to their beliefs (*r*_*s*_ = 0.16, *p* = .017) and their attitudes (*r*_*s*_ = 0.25, *p* < .001) regarding cooperative learning methods. Inversely, as expected, the relative adherence to ST values was negatively linked with their beliefs (*r*_*s*_ =  −0.19, *p* = .003) and their attitudes (*r*_*s*_ =  −0.31, *p* < .001) regarding competitive learning methods.^7^

### Discussion

Our findings corroborated previous research by highlighting the centrality of ST values among individuals choosing a career in teaching (e.g., Ros et al., 1999). The results also showed that pre-service teachers exhibited more positive beliefs and attitudes about cooperative methods than about competitive methods. As predicted, ST value adherence was positively associated with cooperative learning and negatively with competitive learning, both in terms of beliefs and attitudes. Hence, relying on these findings and Schwartz’s model of values, this study also supports the argument that ST values may constitute a motivational basis of individuals’ teaching beliefs and attitudes regarding cooperative and competitive methods. Yet, we note that the strength of the relationships between ST values and cooperative variables was only small to moderate. A plausible reason for the deflated correlations could be the low variability scores (Goodwin & Leech, 2006), which were probably caused by social desirability bias. Social desirability might occur if participants intended to portray themselves as more favourable to cooperation to gain social approval.

In summary, this study demonstrates that the relative adherence to ST values is positively related to attitudes and beliefs regarding cooperative methods. However, since ST values are strongly endorsed by the teaching population, one might expect that cooperative methods would also be widely used in classrooms. Yet, as we have already argued, this is not the case. To explain this paradox, we postulated that the expression of ST personal values might be hindered by the presence of conflictual contextual values (SE values). Thus, our goal in the following studies was to investigate the role of contextual values in the relationship between ST values and cooperative learning methods (in terms of attitudes/beliefs). Contextual values were experimentally manipulated to emphasise either SE values, ST values, or none of the values (control condition).

## Study 2

### Method

#### Participants/procedure

One hundred and twenty-three students (77.05% women, *Mage* = 22.69, *SD* = 5.25) attending an educational sciences course^8^ of a French-speaking Swiss university voluntarily participated in the study.^9^ Students were informed that the purpose of the study was to examine their opinions regarding the economic and educational system and individuals role in society. They were also explained that their participation was anonymous. After having completed the questionnaire, respondents were collectively debriefed and thanked for their participation.

#### Measures

##### Personal values

Participants first completed the measure of personal values. As in the previous study, we calculated the relative adherence to ST values (α = .82) using the following formula: ST values = individual raw mean of ST value items—individual mean score for all value items. However, in this study, we used a more recent version of Schwartz’s value scale used in Study 1 ( PVQ-R; Schwartz et al., 2012, translated into French by Pulfrey, Schwartz, Crouzevialle, and Butera, 2017). This updated version corresponds to the same theoretical model of values with the difference that it also enables researchers to assess subdimensions of each type of value. However, we note that our research only focused on the high-order categories of values. Each item of the scale consists of a short description of important aspects in the life of different hypothetical individuals, reflecting one of the different types of values. Participants are invited to report the extent to which the described person is similar to them on a 6-point scale (1 “not at all like me” to 6 “completely like me”). An example of an item corresponding to ST values was, “It is important to that person that every person in the world is treated equally.”

##### Manipulation of values conveyed by the context

After completing the personal value questionnaire, participants were introduced to the experimental manipulation of the contextual values (see Appendix 1). Specifically, they were instructed to read an extract from a speech on what makes a “good” economy, ostensibly given by an expert, a Nobel Prize winner in economic sciences (see Pulfrey & Butera, 2013). The economist’s conception of the “good” economy varied among the three conditions. In the first condition (ST condition, N = 41), the speech presented the model of a good economybased on ST values. It highlighted the importance of helping others, protecting the environment, being loyal and broad-minded, and promoting social justice and equality. In the second condition (SE condition, N = 43), the model was presented as being  founded on SE values, hightlighting the importance of being successful, having power, authority, and wealth, and maintaining social recognition and a positive image. In the third condition (control condition, N = 39),the model of good economy was presented in very general and abstract terms, without any reference to any category of values. To ensure that participants had read the text, we also asked them to answer a series of questions regarding its content.

##### Beliefs and attitudes regarding cooperative learning methods

Participants were then asked to report their beliefs (α = .65) and attitudes (α = .70) regarding cooperative learning methods. The same scale was used as in Study 1. However, because our sample consisted of students in educational studies, but with different career orientations, the wording of the questions was slightly revised and adapted to be meaningful for all participants.^10^

### Results

Our hypothesis suggested that there should be a positive link between relative adherence to ST values and beliefs about cooperative methods in the ST condition and the control condition, but that this relation should disappear in the SE condition. According to these specific predictions, the three experimental conditions were broken down into two planned contrasts. The first contrast tested our hypothesis (contrast 1): the SE condition (coded − 2) was compared to the control (coded 1) and the ST conditions (coded 1). The second orthogonal contrast (contrast 2) compared the ST condition (coded 1) to the control condition (coded − 1), with the SE condition in the middle (coded 0). To reject the null hypothesis the contrast testing our hypothesis (contrast 1) should be significant, and the orthogonal contrast (contrast 2) should be nonsignificant (Judd et al., 2011).

Thus, a regression model (with robust standard errors) included the main effect of relative adherence to ST values, the two contrasts and the interaction effects between ST value adherence﻿ and the two contrasts. Continuous variables included in the interaction in the current and in all the following studies were standardised.

#### Beliefs about cooperative learning methods

**Fig. 1:**
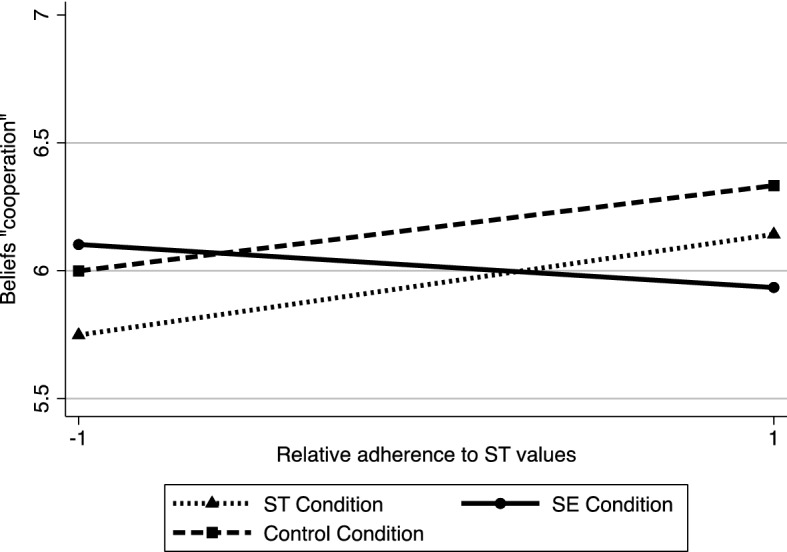
Interaction between relative adherence to ST values and context portraying SE values, ST values, and control on beliefs about cooperative learning (Study 2)

Regression diagnostics showed the presence of one influential case (Cooks Distance = 0.10). This case was eliminated from this analysis. Results revealed that none of the main effects were significant (ST values, *b* = 0.09, *SE* = 0.06, *t*(116) = 1.59, *p* = .115, 95% CI [− 0.02, 0.21], η^2^ = 0.02; Contrast 1, *b* =  −0.01, *SE* = 0.04, *t* =  −0.31, *p* = .755, 95% CI [− 0.10, 0.07], η^2^ = 0.0008; Contrast 2, *b* =  −0.11, *SE* = 0.07, *t*(116) =  −1.58, *p* = .118, 95% CI [− 0.25, 0.03], η^2^ = 0.02). In line with our hypothesis, results showed that the interaction between the relative adherence to ST values and the contrast 1 was significant, *b* =  −0.09, *SE* = 0.04, *t*(116) =  −2.00, *p* = .049, 95% CI [− 0.18, − 0.0004]), *η*^2^ = 0.03 (see Fig. 1),indicating that the link between ST value adherence and beliefs regarding cooperative learning methods was different in the SE condition compared to the other two conditions. The interaction between the contrast 2 and ST value adherence was not significant(*b* = 0.01, *SE* = 0.07, *t*(116) = 0.23, *p* = .822, 95% CI [− 0.12, 0.15], η^2^ = 0.0005).

Analysis of simple effects of the significant interaction showed that, in line with our predictions, adherence to ST values positively predicted beliefs about cooperative methods in the ST condition and control condition (combined effect, *b* = 0.18, *SE* = 0.07, *t*(116) = 2.75, *p* = .007, 95% CI [0.05, 0.31], η^2^ = 0.06), but not in the SE condition (*b* =  −0.08, *SE* = 0.11, *t*(116) =  −0.72, *p* = .472, 95% CI [− 0.31, 0.15]), η^2^ = 0.004).

#### Attitudes towards cooperative learning methods

**Fig. 2:**
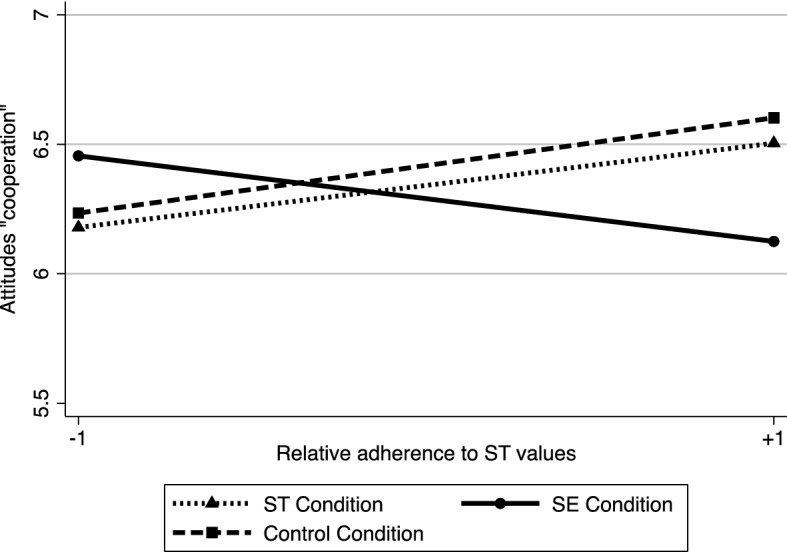
Interaction between relative adherence to ST values and context portraying SE values, ST values and control on attitudes towards cooperative learning (Study 2)

The same regression model with robust standard errors was conducted on the attitudes towards the use of cooperative learning methods. Again, we kept the same influential case out of this analysis (Cooks Distance = 0.10). Results did not reveal any significant main effects (ST values, *b* = 0.06, *SE* = 0.05, *t*(116) = 1.20, *p* = .233, 95% CI [− 0.04, 0.16], η^2^ = 0.01; Contrast 1, *b* =  −0.03, *SE* = 0.04, *t*(116) =  −0.84, *p* = .405, 95% CI [− 0.11, 0.04], η^2^ = 0.006; Contrast 2, *b* =  −0.04, *SE* = 0.06, *t*(116) =  −0.62, *p* = .538, 95% CI [− 0.16, 0.08], η^2^ = 0.003). The predicted interaction between the ST values adherence and the first contrast (contrast 1) was significant (*b* =  −0.11, *SE* = 0.04, *t*(116) =  −3.05, *p* = .003, 95% CI [− 0.18, − 0.04], η^2^ = 0.07; see Fig. 2). Contrarily, the second interaction term, the contrast 2*ST value adherence, was not significant (*b* =  −0.01, *SE* = 0.06, *t*(116) =  −0.18, *p* = .858, 95% CI [− 0.13, 0.11], η^2^ = 0.0003). Analysis of simple slopes of the significant interaction showed that the link between ST values and attitudes towards cooperative methods was positive in the ST condition and the control condition (combined effect, *b* = 0.17, *SE* = 0.06, *t*(116) = 2.93, *p* = .004, 95% CI [0.06, 0.29], η^2^ = 0.07), but negative and marginally significant in the SE condition (*b* =  −0.16, *SE* = 0.09, *t*(116) =  −1.76, *p* = .081, 95% CI [− 0.35, 0.02], η^2^ = 0.03).

### Discussion

In line with our predictions, we observed that the effect of personal (relative) adherence to ST values on beliefs/attitudes regarding cooperative methods varied across different value contexts. While the effect was positive and did not differ between the context conveying ST values and the value-neutral context (providing results similar to those observed in Study 1), it was null(or became negative) within the context conveying SE values. Hence, the presence of conflictual values within a specific environment or a situation, might have acted as a countervailing force, diminishing individuals’ value-expressive beliefs and attitudes.

In this study, our paradigm explicitly manipulated contextual values at a macro-level, quite distal from a specific classroom situation. This enabled us to demonstrate the influence of externally promoted values that refer to the broader societal level, on individuals’ value-expressive responses. However, as the values in this study were rendered salient through a discourse made by an expert authority, it s possible that the legitimacy of this source (rather than the content of values per se) had influenced participants’ answers, by leading them to demonstrate consistency with the former (French & Raven, 1959; Raven, 1993). To rule out this possibility, in the next study, contextual values were implicitly manipulated without reference to any source. If our hypothesis is valid, the impact of contextual values should be independent of the specific characteristics of means and the level at which they occured. We argued that SE values are chronically accessible within educational settings in Western societies and, thus, could be easilyretrieved from the memory, even with an implicit activation, resulting in an influence on individuals’reactions (Bargh et al., 1986). Therefore, using a priming technique, we tested whether an implicit activation of contextual values could also moderate the relationship between individuals’ values and beliefs.

## Study 3

### Method

#### Participants/procedure

One hundred and five students of educational sciences (82% women) agreed to participate in the study during a course at a French-speaking Swiss university. The majority of our sample (93.33%) was in the first year of their studies. The first year is common to all students in the educational sciences, independent of their orientation in the second year. Two individuals (1.90%) were in the second year of their training in primary teaching, and five (4.76%) individuals had different orientations.

Participants were asked to complete a questionnaire designed to investigate two unrelated research objectives. They were told that the objective of the first part was to find out their opinions about different aspects related to society and the educational system. This involved the measurement of personal values. The bogus objective of the second part was to elicit their propositions regarding teaching material for pupils. More precisely, they were told that we were developing teaching material on the narrative text (a disciplinary topic in primary schools) and that we would like their contribution to its construction. All students in the class agreed to participate. Since the experiment was conducted at the end of the course, time was limited, and we were not able to measure their attitudes towards cooperation. At the end of the questionnaire, participants were given a written debriefing.

#### Measures

##### Personal values

First, participants answered a shortened version of the same scale used in Study 2 (PVQ-R; Schwartz, et al., 2012). The shorter version contained 31 items and was preferred from the extended original version due to time constraints. The relative adherence to ST values (α = .76) was calculated as in the previous studies (ST values = individual raw mean of ST items—individual mean score for all value items).

##### Manipulation of values conveyed by the context

Subsequently, the experimental manipulation was introduced. Participants were exposed to a series of four images that differed among the three conditions to which they were randomly assigned. They were all instructed to write a story based on these images (using at least three of the four presented), which could be used as an example for 2nd grade pupils in primary school. They were also requested to assign a title to their story. In the first condition, participants viewed images that referred to ST values (N = 32, ST condition). In the second condition,  images referred to SE values (N = 34, SE condition). In the third condition (N = 39, control condition) images were neutral without links to any types of value. The images in the ST and SE conditions were taken from the validated children’s value scale (Döring, 2010). The images in the control condition were taken from a children’s book and had a similar format to the images in the other two conditions.

##### Beliefs regarding cooperative learning methods

Participants’ beliefs about cooperative learning methods (α = .62) were measured with the same scale as in Study 2.

### Results

#### Beliefs about cooperative learning methods

**Fig. 3:**
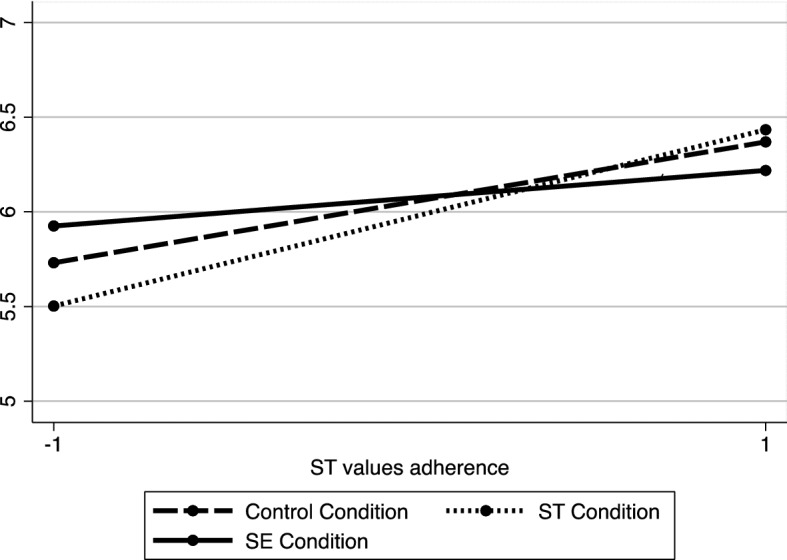
Interaction between relative adherence to ST values and context portraying SEvalues, ST values and control on beliefs regarding cooperative learning (Study 3)

The same regression model was run as in the previous study. We report simple standard errors, as regression diagnostics did not show any special normality issues of residuals. Results showed a main effect of ST value relative adherence (*b* = 0.31 *SE* = 0.06, *t*(99) = 5.33, *p* < .001, 95% CI [0.19, 0.42], η^2^ = 0.22) on beliefs about cooperative methods. Neither the main effect of contrast 1 (*b* = 0.02, *SE* = 0.04, *t*(99) = 0.51, *p* = .608, 95% CI [− 0.06, 0.10]) nor that of contrast 2 (*b* = 0.04, *SE* = 0.07, *t*(99) = 0.67, *p* = .554, 95% CI [− 0.10, 0.18], η^2^ = 0.005) was significant. Similar to the previous study, we observed a significant interaction between contrast 1 and ST value adherence (*b* =  −0.08, *SE* = 0.04, *t*(99) =  −2.13, *p* = .036, 95% CI [− 0.16, − 0.005], η^2^ = 0.04) indicating that, consistent with our hypothesis, the link between ST value adherence and beliefs regarding cooperative learning differed in the SE condition compared to ST and control conditions (Fig. 3). The interaction between the contrast 2 and the ST value adherence was not significant (*b* =  −0.07, *SE* = 0.08, *t*(99) =  −0.97, *p* = .336, 95% CI [− 0.22, 0.08], η^2^ = 0.009). The examination of simple effects of the significant interaction revealed that the link between ST value adherence and beliefs regarding cooperative learning was positive and significant in both the control and ST conditions (combined effect, *b* = 0.39, *SE* = 0.07, *t*(99) = 5.14, *p* < .001, 95% CI [0.24, 0.54], η^2^ = 0.21), but not in the SE condition (*b* = 0.14, *SE* = 0.09, *t*(99) = 1.62, *p* = .108, 95% CI [− 0.03, 0.31], η^2^ = 0.03).

### Discussion

This study showed that an implicit activation of SE values in the context (i.e., without any source producing an explicit influence message) could erode the positive relationship between relative adherence to ST values and beliefs regarding cooperative learning methods. In contrast, within an ST value-activated, or a value-neutral context, individuals’ adherence to ST values positively predicted beliefs regarding cooperative learning methods.

These results complement those of the previous study, by showing that SE values undermined the expression of ST personal values, not only when they were explicitly emphasised by an authority, but also when they were implicitly salient in the teaching material. Importantly, these findings indicate that the influence of contexualvalues not only occurs when these values are portayed in socio-economic terms at the distal, societal level (Study 2), but also when done so at a more proximal level in an individual’s social space. As we observed, contextually significant values can be evoked through artefacts in the specific context of the classroom.

So far, we have investigated the effect of the interaction between personal and contextual values on beliefs and attitudes regarding cooperative methods. Indeed, beliefs and attitudes regarding teaching methods, in general, play a considerable role when we attempt to understand teachers’ instructional orientations in classroom (Maxwell, 2015; Richardson, 2003). However, our ultimate goal is to understand teaching behaviours. Therefore, in the next study, we aimed to replicate the results of the two previous studies using a behavioural outcome that is more concrete. To do so, pre-service teachers were invited to read a text describing a plausible scenario in a primary school and asked to report to what extent they would use cooperative methods for their pupils. Thus, in keeping with the aim to approach teachers’ reality at work, contextual values were manipulated at the school level. Having confirmed the effect of contextual values at both a macro and micro level, extending these results at a meso-level would not only constitute a valuable addition in the research results but could also identify a greater number of possibilities and means for intervention.

Values relevant in a school are conveyed by its rules, goals, strategies and the activities it tries to promote (Daniel et al., 2013; Friedman, 2003; Tal & Yinon, 2009). Hence, each school, as an organisation, is expected to have its value orientation underlying school culture. School values may have a prescriptive role, implying that its members may feel obliged to align their values with the school’s value culture (Pang, 1996). For instance, indirect empirical evidence has shown that a school’s administration may hinder teachers’ autonomy by exerting pressure on their instructional behaviours (Pelletier & Sharp, 2009). Study 4 investigated whether a school context emphasising different types of values (ST values vs. SE values vs. control) has an impact on the positive relationship between individuals’ ST values and their willingness to use cooperative methods.

## Study 4

### Method

#### Participants/procedure

An invitation to participate in this study was sent to all pre-service teachers that were either in the final or in the pre-final year of their training at a French-speaking Swiss university (57.89% pre-final year, 42.11% final year of their studies). Eighty students (*M*_age_ = 26.12, *SD* = 6.29; 81.58% women) agreed to voluntarily participate in this study, which was carried out over the Internet. Participants were told that  the aim of the sudy was toexplore their opinions about different aspects of society and the educational system. At the end of the questionnaire, participants were given a written debriefing.

#### Measures

##### Personal values

Similar to the previous two studies, we initially measured personal values with Schwartz’s refined values scale (PVQ-R, Schwartz et al., 2012). The full-length scale was used, and the relative adherence to ST values was calculated as described in previous studies (ST values = individual raw mean of ST items—individual mean score for all value items).

##### Manipulation of values conveyed by the context

Participants were then asked to imagine that they had just been hired as 3rd grade teachers in a public primary school in Switzerland. They were instructed to read a speech that was supposed to be given by the principal of the school in which they were going to work. They were also told that the aim of this speech was to present the mission, vision, and values of the school. The content of the speech varied among the three experimental conditions to which they were randomly assigned (see Appendix 2 for details of the text). More precisely, in the SE condition (N = 25), the speech presented a school in which SE values were encouraged. This school was presented as having the mission of promoting excellence and the development of ambitious educational projects. It was also highlighted that values of personal achievement, ambition, and power were essential for preserving a competent community and a positive image. In the ST condition (N = 29), the school was presented as having a mission to develop tolerance and educational projects based on the mutual aid of its members. Values of equality, mutual respect, and social justice were essential for ensuring a benevolent community, a harmonious environment, and the growth of all. In the control condition (N = 26), the speech presented the mission of the school in general and abstract terms, with no reference to any type of values.

##### The choice of cooperative learning methods 

Unlike the previous studies focusing on attitudes and beliefs, in the current study the outcome variable referred to a behavioural intention. After reading the speech, participants were asked to report the extent to which they would have chosen to implement cooperative methods as 3rd grade teachers in the designated school. A list of different instructional methods along with a short definition (e.g., transmissive teaching, collective discussion, individual work, or tutoring) was presented to participants. Cooperative group work was also included in this list. Participants were asked to allocate 100 points (a total score of 100%) among the different methods in a way that represented how they would organise pupils’ learning in their classroom.

The instructional methods were negatively interdependent, and a higher allocated score in a method expressed a greater willingness to implement this method relative to the other listed alternatives. Participants were also allowed to add supplementary instructional methods that were not listed (only a small minority chose to do so). We included different instructional methods, without exclusively focusing on cooperative methods, mainly because we wanted to attenuate social desirability bias by making the research objective less visible. Moreover, we believed that the negative interdependence between the different instructional methods better represented the real-life situation in the classroom where teachers prioritise some instructional methods over others.

### Results

#### The choice of cooperative learning methods

Since our outcome variable was in ratio(percentages), and thus may not respect normality assumptions of a linear regression model, we conducted a fractional regression model (probit) with robust standard errors with the same contrast analysis strategy^11^including the same terms in the equation as in the previous studies. Results revealed a main effect of the ST values (*b* = 0.10, *SE* = 0.03, *z*(75) = 3.44, *p* = .001, 95% CI [0.04, 0.15], η^2^ = 0.15), indicating that more participants prioritise ST values the more they chose cooperative group work. Contrast 1, which compared the SE condition to the other two, was not significant (*b* =  −0.03, *SE* = 0.02, *z*(75) =  −1.24, *p* = .213, 95% CI [− 0.08, 0.02], η^2^ = 0.02). Neither was contrast 2, which compared the ST condition to the control condition (*b* = 0.05, *SE* = 0.04, *z*(75) = 1.30, *p* = .193, 95% CI [− 0.03, 0.13], η^2^ = 0.02). The interaction testing indicated our hypothesis did not reach the level of significance for this study, but the obtained results went strongly in the same direction as the preceding ones (*b* =  −0.03, *SE* = 0.02, *z*(75) =  −1.40, *p* = .16, 95% CI [− 0.07, 0.01], η^2^ = 0.03). Moreover, the interaction between contrast 2 and relative adherence to ST values was not significant (*b* = 009, *SE* = 0.03, *z*(75) = 0.28, *p* = .783, 95% CI [− 0.05 0.07], η^2^ = 0.001).

**Fig. 4:**
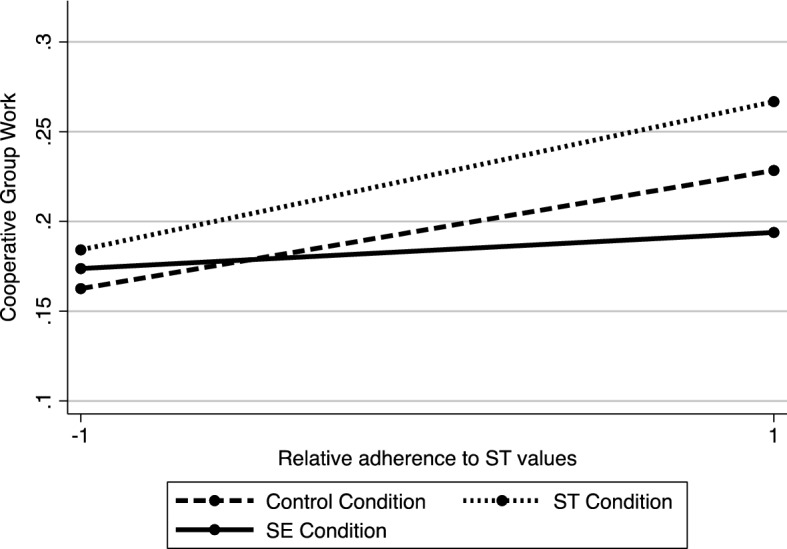
Interaction between relative adherence to ST values and school portraying ST values,SE values,  and control on the choice of cooperative group work (Study 4)

Despite the fact that these results indicated no difference between SE and the other two conditions, we decided to decompose the interaction to test whether the simple effects were consistent with our hypothesis and our previous findings in Studies 2 and 3 (Fig. 4). Analysis of simple effects showed that, as in previous studies, the link between ST values and the choice of cooperative work was positive and significant in the combined ST and control condition (*b* = 0.04, *SE* = 0.01, *t*(74) = 3.84, *p* = .003, 95% CI [0.02 0.06], η^2^ = 0.18), but it was not significant in the SE condition (*b* = 0.01, *SE* = 0.01 *t*(74) = 0.67, *p* = .503, 95% CI [− 0.02 0.04], η^2^ = 0.006).

### Discussion

This study tested the hypothesis that the school value orientation moderates the relationship between pre-service teachers’ ST value adherence and their intention to use cooperative group work among their future pupils. Although the results yielded a similar pattern to those from the two previous studies, they did not reach the threshold of statistical significance. However, the effect size of this interaction was similar to that observed in the two previous studies. It is therefore likely that the absence of statistical significance was due to our small sample size^12^ (Durand, 2013) and its unsufficient power to detect such interactions (Toothaker et al., 1994). Nonetheless, because an invitation to participate in this study was addressed to all candidate primary school teachers enrolled in the teaching training, it was practically difficult to gain more precision by increasing our sample size.

The following study aimed to extend previous results on teachers’actual use of cooperative learning methods. It investigated whether perceived SE values in the school environment attenuate the link between teachers’ ST value adherence and the self-reported frequency of using cooperative methods. A recommended method for measuring contextual values (e.g., Abbott et al., 2005; Fischer, 2006) involves relying on individuals’ perceptions of the importance of each value in an organisation. Thus, in the following study, school values were captured by teachers’ perceptions of what was valued in their school (Jourdain & Chênevert, 2015).

## Study 5

### Method

#### Participants/procedure

A total of 161 in-service teachers (88.83% women, *M*_*a*ge_ = 41.26, *SD* = 9.94) working in different primary schools in French-speaking Switzerland agreed to participate in this study over the Internet. To reach teachers, we contacted school principals of primary schools in the area. The principals were given information about the purpose of the study and its modalities and were provided with a link to the questionnaire online. Most of them agreed to transfer the invitation to teachers working in their school. The study was presented as having the goal of exploring teachers’ opinions about aspects that are important in their lives and their school and about different instructional practices implemented in classrooms. Participation consisted of anonymously answering a series of self-reported questionnaires.

#### Measures

##### Teachers’ adherence to ST values

First, participants’ relative adherence to ST values (*M*_raw score_ = 5.34, *SD* = 0.43, *M*_relative score_ = 0.99, *SD* = 0.33) was measured with the same adapted scale of Schwartz’s revised value questionnaire used in Study 3 (PVQ-R, Schwartz et al., 2012). The shortened version was preferred due to time limitations. As in our previous studies, to calculate relative adherence to ST values (α = .76), all four high-order categories of values were measured (ST values = individual raw mean of ST items—individual mean score for all value items).^13^

##### Perceived school relative adherence to SE values

To measure a school’s relative adherence to SE values (*M*_raw_ score = 2.61, *SD* = 0.74, Min = 1.14, Max = 4.86; *M*_relative score_ =  −1.62, *SD* = 0.76), we asked participants to complete an identical scale to the one measuring their personal values. However, this time, their answers should represent what is important within their school (the wording of the items was adapted to be meaningful for measuring school values). More precisely, it was first explained that as organisations, schools, can be responsible for defining school objectives and undertaking actions to fit the local context and meet specific needs. With this in mind, participants were asked to indicate the extent to which each of the descriptions (items) referring to one of the four categories of values, was reflected in the undertaken actions of their school. Each description started with the following expression: “It is important in my school…”. For instance, an item corresponding to SE values was “It is important in my school to have power and to impose decisions”. An item corresponding to ST values was “It is important in my school that all people are treated in an equal way”. All items were rated on a 6-point scale. Again, to assess the extent to which participants perceived that their school adheres to SE values (SE school, α = .77) relative to all values (relative adherence), we measured the perceived school adherence to all four categories of values. We then calculated the mean of all value items and we subtracted it from the corresponding mean of SE values.

##### Grade level

In the current study, the grade level at which participants were teaching (from preschool to 6th grade) was included in the model as a control variable. Previous research (Kyndt et al., 2013; Lou et al., 1996) has shown that the success of cooperative–method implementation may depend on the grade level. We argue that it is also possible to influence the frequency with which teachers propose these methods in their classrooms.

##### Frequency of the implementation of cooperative methods

Respondents were then asked to report on an ordinal scale of five points (rarely, occasionally, in moderation, regularly, often) the frequency with which they usually (in a typical teaching week) implement cooperative group work in their classroom. As in the previous study, a definition of cooperative group work was also provided to ensure all participants had a similar understanding of this method (i.e., pupils work in small groups with a common objective for the team). Scale distribution was normal, and this variable was treated as a continuous variable.

### Results

The regression model contained teachers’ perceived school relative endorsement of SE values, teachers’ relative adherence to ST values, the grade level, and the interaction terms between all the above variables (school SE values* teachers’ ST values*Grade level; school SE values* teachers’ ST values; teachers’ ST values*Grade level; school SE values* Grade level). An influential observation was found and removed from the data (Cook’s Distance = 0.76). Since residuals were not normally distributed (Shapiro–Wilk test = 2.67), we conducted a regression analysis with robust standard errors.

**Fig. 5:**
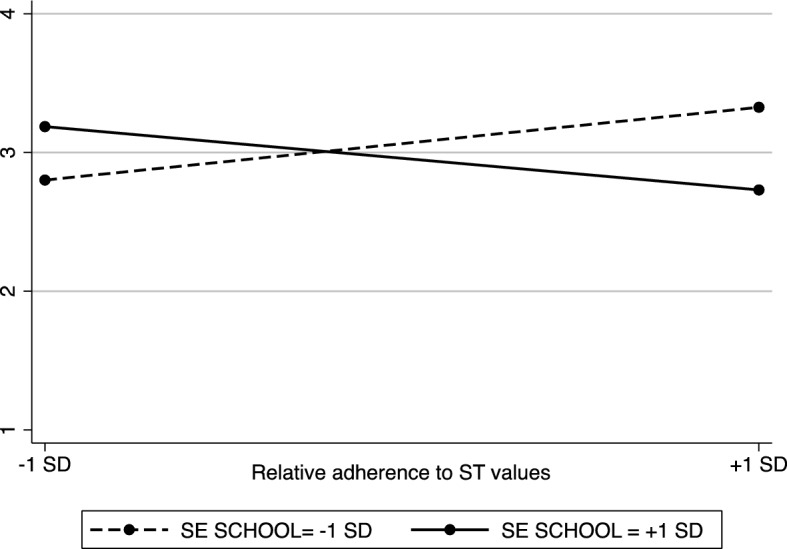
The interaction effect between teachers' (relative) adherence to ST values and the perceived school (relative) adherence to SE values on the frequency of cooperative group work implementation (Study 5)

Results indicated the interaction (Fig. 5) between teachers’ relative adherence to ST values and their perceptions about school SE value adherence was significant (*b* =  −0.22, *SE* = 0.09, *t*(152) =  −2.57, *p* = .011, 95% CI [− 0.39 − 0.05], *η*^2^ = 0.04). An analysis of simple slopes showed the relationship between teachers’ relative adherence to ST values and the frequency of cooperative work implementation was positive and significant when the school was perceived to weakly (- 1 *SD*) endorse SE values, (*b* = 0.26, *SE* = 0.12, *t*(152) = 2.14, *p* = .034, 95% CI [0.02, 0.50], η^2^ = 0.03), but was insignificant when the school was perceived to strongly endorse (+ 1 *SD*) SE values (*b* =  −0.23, *SE* = 0.14, *t*(15) =  −1.63, *p* = .105, 95% CI [− 0.50, 0.05], η^2^ = 0.02). The main effect of the grade level was also significant, indicating that the frequency of cooperative group methods application, increased with an increase in grade level (*b* = 0.21, *SE* = 0.09, *t*(152) = 2.18, *p* = .031, 95% CI [0.02, 0.39], η^2^ = 0.03). The main effects of teachers’ ST value adherence (*b* = 0.004, *SE* = 0.09, *t*(152) = 0.05, *p* = .963, 95% CI [− 0.17 0.18], *η*^2^ = 0.00) and the perceived school SE value adherence (*b* =  −0.04, *SE* = 0.10, *t*(152) =  −0.32, *p* = .747, 95% CI [− 0.73 0.17], η^2^ = 0.00) were not significant. None of the interactions between teachers’ ST values, school SE values, and the grade level were significant (teachers' ST values *SE school values*Grade level, *t*(152) = 1.45, *p* = .148; SE school values *Grade level, *t*(152) = 1.26, *p* = .212; teachers’ ST values*Grade level, *t*(152) =  −0.80, *p* = .423).

### Discussion

This study revealed that the degree to which teachers perceived their school to endorse SE values moderated the positive link between their relative adherence to ST values and their reported use of cooperative methods in the classroom. More precisely, our results showed that teachers acted consistently with their personal values, only when they perceived their school to attribute low importance to SE values. In contrast, when they perceived that their school attributed high importance to SE values, their relative adherence to ST values did not predict the use of cooperation. These findings seem to corroborate previous results by expanding the hypothesis regarding the moderating role of contextual values that conflict with individual values in behavioural outcomes. To our knowledge, this study is the first attempt to examine how perceived school value culture contributes to the link between personal values and teaching practices.

To attain greater statistical power, in the next section, we report on an internal meta-analysis aggregating the observed effect in this study with the effects in the three previous studies (Stukas & Cumming, 2014). If the summary of this evidence differs from zero, we can add complementary evidence in favour of our hypothesis.

## Meta-analysis of Studies 2, 3, 4, & 5

An internal meta-analysis (Bender et al., 2018) was conducted on Studies 2, 3, 4, and 5 to summarise their effects and test whether there was consistency and sufficient evidence to support our hypothesis, suggesting that the more individuals prioritise ST values, the more they will be in favour of cooperation as a learning strategy (in terms of beliefs, attitudes, intentions, and reported behaviours) only when SE values are not emphasised within the context.

We performed a meta-analysis using fixed effects^14^ in which the mean effect size of the hypothesised interaction^15^ was weighted according to the sample size of each study. We note that because we had two dependent variables in Study 2 and the effects sizes should be independent, we averaged the effect sizes of each dependent variable to have one effect size per study (Goh et al., 2016).

**Fig. 6:**
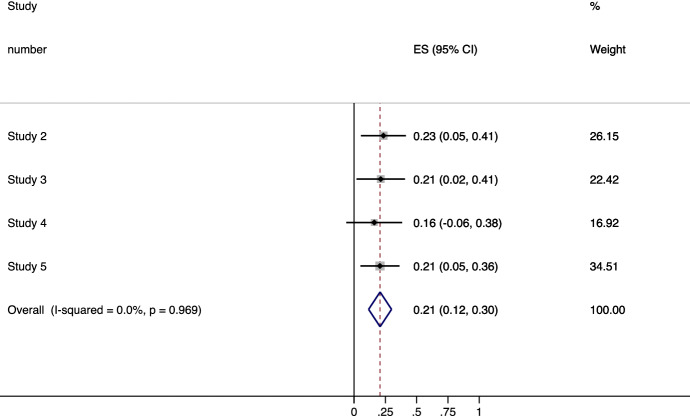
Forest plots of the internal meta-analysis of studies 2, 3, 4, 5, for the effects of the interaction contrast 1*individuals’ relative adherence to ST values, on outcomes regarding cooperative learning methods

Thus, we first calculated the partial effect size *r* (Aloe & Thompson, 2013) and we applied a Fischer’s z scale transformation to all effect sizes. Results showed that across the four studies, the average effect size was significant and there was homogeneity (Fig. 6) (*Pooled Effect Size*, *Mz* = 0.21, z-test,^16^*z* = 4.43, *p* < .001, 95% CI [0.12 0.30], Cohen’s *d* = 0.43, heterogeneity, χ^2^(3) = *0.2*5, *p* = .968).

For Studies 2, 3, and 4, in which our interaction hypothesis was tested with contrast analysis (i.e., experimental conditions were broken down in two orthogonal contrasts), it was also necessary to confirm that the interaction between the residual contrast 2 and the relative adherence to ST values was not significant. Therefore, the same weighted procedure was conducted among the effects on the second interaction (*Pooled Effect Size*, *Mz* = 0.049, *z* = 0.84, *p* = .399, 95% CI [− 0.07 0.16], heterogeneity, *χ*^2^(2) = *0.2*7, *p* = 0873). Overall, results of this meta-analysis seemed to support our hypothesis, suggesting that the link between ST values and cooperation differs between SE value-context and a ST value-context or a neutral context.

## General discussion

This research attempts to offer a deeper understanding of the paradox between the teachers’ positive viewsregarding cooperative learning methods (Johnson et al., 2000; Ruys et al., 2010; Slavin, 2014) and their marginal use of such methods in classrooms (Baines et al., 2003; Buchs et al., 2017; Pianta et al., 2007; Wasik, 2008). We approached this recurring phenomenon by investigating a hypothesis that integrates both individual-level and contextual-level factors. More precisely, we aimed to understand whether a clash between personal values and contextual values affects beliefs, attitudes and behaviours regarding the use of cooperative learning methods. Studying a sample of pre-service teachers, we first demonstrated that ST values are generally strongly endorsed by those intending to pursue a career in teaching, which corroborates results of previous studies (Ros et al., 1999; Vansteenkiste et al., 2006). Additionally, we observed that the relative importance attached to ST values was positively related to teachers’ beliefs and attitudes regarding cooperative learning methods. The endorsement of ST values and its positive association with cooperative methods could be a powerful argument to explain the prevailing positive views towards these methods among professionals in the field of education (Ruys et al., 2010; Tal, 2018). Yet, if teachers—driven by their personal values—are inherently motivated towards cooperative learning methods, then why are these methods underused in classrooms?

To answer this question, we investigated whether the presence of conflicting contextual values would diminish the positive link between ST values and cooperative methods. A set of four studies yielded the same pattern of results: When the context emphasised SE values, ST personal values were no longer linked with cooperative outcome variables. Our findings provide strong support for this hypothesis for several reasons. the hypothesised effect was confirmed under a number of different circumstances: When activation of SE values was either distal or proximal to the classroom context, when the values were either explicit or implicit, among three different populations (students enrolled in educational sciences programs, pre-service teachers and in-service teachers) and when using different methodological orientations (i.e. contextual values were either measured or manipulated). In addition, this clash between values was found to affect both cognitive and affective aspects of individuals’ mental states—their beliefs and attitudes, respectively—and, more importantly, actual or intentional cooperative learning use. Thus, these results suggest that values are broadly linked with individuals’ perspectives and implementation of cooperative methods.

This research offers a significant contribution to scientific literature on cooperative learning instruction. Attempting to explain low use of cooperative methods in classrooms, previous research has mainly focused on teachers’ professional characteristics (Gillies & Boyle, 2010) and practical difficulties related to the implementation of such methods (Buchs et al., 2017; Ruys et al., 2014; Tal, 2018). Overall, much of the existing research revealed demonstrates that implementing cooperative methods may be a challenging task for teachers, as it requires specific training, professional competence, staff coordination and adequate classroom management. Our results complement this literature by providing evidence that teachers’ personal values may also have a determinant role. They lend support to the argument raised in previous studies (e.g. Tal, 2018) about the significant role of teachers’ motivation for ensuring an efficient, long-term use of cooperative learning. Our study illustrates that motivational underpinnings of the use of cooperative methods may stem from adherence to ST values, whose expressions are conditional to the absence of conflicting SE values in the context.

As we have argued, SE values are particularly emphasised in educational institutions in Western societies, where they express cultural and school expectations (Branco, 2018). Thus, their prescriptive role is likely to be chronically accessible and internalised by individuals throughout their education and career. Similarly, the present results show that SE values affect individuals at different stages of their careers. We found significant differences between students in educational sciences programs, pre-service teachers and in-service teachers. For example, Study 5 found that participants’ perceived SE value endorsement in the school environment varied from *M*_min_ = 1.14 to *M*_max_ = 4.86 on a 6-point scale, indicating that some teachers perceive SE values as important within their school. 

Another significant aspect of our work is that it may offer an important perspective for better understanding results observed in the two meta-analyses, which demonstrated that cooperative learning methods were less effective within Western cultures (Balta et al., 2017; Kyndt et al., 2013) in which SE values were emphasised than within collectivist ones (Kasser et al., 2007; Sagiv & Schwartz, 2007). Our findings also corroborate previous educational research showing that perceived contextual pressures can restrain teachers from acting according to their own personal preferences (Hornstra et al., 2015; Pelletier & Sharp, 2009; Ruys et al., 2014; Tal & Yinon, 2002).

Furthermore, the robust empirical support on the interaction between individual-level and contextual-level values may also constitute an essential contribution to the research of values. It primarily supports scholars’ recommendations that stress the importance of considering social context values to understand under which conditions personal values can guide individuals’ lives (Bardi & Schwartz, 2003; Roccas & Sagiv, 2017). Importantly, our findings further extend this argument by highlighting that values arise at different levels within a context—in this instance, at a micro (teaching material), meso (school values) to macro-level (society)—and can be activated explicitly or implicitly, thereby interacting with an individual’s values at any level.

From an applied perspective, this work also provides useful insights for favouring cooperative learning implementation. Indeed, it may be more difficult to inhibit SE values at the macro level of society or at the level of school institutions, as these are expressed mainly through policies and practices underlying educational standards at the national and international levels. Instead, an individual may more easily act at a meso level, that of the school environment. School principals can favour cooperative learning methods in the school by providing trainings and promoting activities, and by implementing a school organisation that supports cooperation. Furthermore, teachers, as members of the school, might also find ways to reduce the strength of SE values. The promotion of teacher collaboration and dialogue could be one of several potential ways to create an environment, propitious to encouraging teachers to act in coherence with their values.

As this research suggests, the discord between contextual and individual values is not only likely to undermine behaviours, but also beliefs and attitudes. If teaching beliefs and attitudes precede teaching behaviours, then it may also be relevant to primarily focus on these two components to promote cooperative methods. Teacher training can take a central place in this endeavour. Because educational programs of teachers’ initial and continuing training often act as the main agents in shaping and challenging teaching beliefs and attitudes, they can contribute to raising teachers’ awareness of the potential of their personal and contextual values on their teaching orientation.

Encouraging professionals to reflect upon their values, beliefs and behaviours may serve as a potential way to challenge and improve their teaching (de Vries et al., 2014). In this view, an in-depth analysis of an individual’s teaching behaviour is very important, as teachers are not always aware of the origins and the effects of their actions. For instance, previous work has highlighted that teachers might sometimes unconsciously promote competition of individual learning among pupils (e.g. comparing pupils’ performance), even when they are instructed to implement cooperation methods (Branco, 2018; Williams & Sheridan, 2010). Given that the promotion of competition and performance goals are firmly anchored within SE school environments, these findings may underscore the implicit influence a normative context may have on teachers. It is therefore crucial for professionals to be aware of this potential impact of values, both personal and contextual, on their teaching.

## Limitations and future directions

Although this research yields robust evidence supporting the hypothesis, some limitations should be acknowledged. The use of self-reported measures is one of the primary limitations of this empirical investigation. Previous work has shown that self-reported measures are sensitive to self-perception and social desirability biases. As we have previously noted, social desirability might be one cause of the low variability observed in our measures, mainly in those related to cooperation, and these could have possibly deflated the strength of the statistical relationships between variables (Goodwin & Leech, 2006). Future research would be strengthened by relying upon different measures for assessing cooperative learning implementation—such as observational methods—that are less sensitive to self-reporting bias. However, social desirability bias may be less problematic for the value measure, as the use of relative (centred) scores instead of their raw scores could be a way to correct for potential social desirability bias (Schwartz et al., 1997). Nevertheless, it would be advantageous for future research to expand upon these results using an implicit measure of values (Souchon et al., 2017).

Another limitation regarding our measurement approach is that in Study 5, both personal values and perceived school value orientation were assessed by the same source (the teachers), at the same time, which could have raised the issue of common variance bias (Podsakoff et al., 2003). Although some authors argue that the issue of common variance bias is exaggerated (Spector, 2006), others propose (Podsakoff et al., 2003) post-estimation remedies, but they may be insufficient to deal with the issue (Antonakis et al., 2014). One of the ways to avoid this potential issue in future studies would be to use instrumental variables (Antonakis et al., 2014) or to measure school values with different source ratings. Overall, reducing potential sources of measurement error (McCartney & Rosenthal, 2000), along with an increase in the sample size (Lovakov & Agadullina, 2021), can be crucial to increase precision in future.

The use of a cross-sectional design in Studies 1 and 5 might also be perceived as a methodological limitation, as it does not allow any causal inferences to be drawn. However, we argue that the diversity in methodologies used may also be viewed as a strength. In other words, the fact that Study 5 confirmed our hypothesis using a different methodological design provides complementary evidence in favour of the robustness of the results. In addition, we believe that experimentally manipulated contextual values—rather than measuring—might hinder capturing the richness of the actual reality within teachers’ professional contexts.

Another point worth noting is that personal values were measured with different value measures. Notwithstanding these different measures were developed on the same theoretical framework (Schwartz, 1992; Schwartz et al., 2012), the differential impact on our results cannot be determined. From an alternate perspective, the replicated pattern of results across studies suggests that our hypothesis holds independently from instrument choice.

Finally, with the aim to better understand the implementation of cooperative methods within the school environment, it could be useful for future research to also account for pupils’ contributions. Indeed, pupils have a determinant role in learning construction, and they could exert an important influence on classroom teaching practices. Assessing how pupils’ personal values interact with contextual values in determining their cooperative behaviours may constitute a fruitful avenue for further understanding the use of cooperative learning in classrooms.

## Conclusion

Taking a comprehensive social-psychological approach, this research considers values at the personal and contextual levels and examines how their interactions may affect cooperative learning methods. At a time when cooperative methods are widely recognised as one of the most promising approaches in learning, our results provide new insights and substantial evidence for understanding their lack of implementation by teachers. The results show a positive relationship between self-transcendence values of benevolence and universalism on cooperative learning, suggesting that teachers’ adherence to these values would support the implementation of these methods in the classroom. However, the results indicate that such a relationship may be context-dependent and hindered by the presence of antagonistic self-enhancement values (i.e. power and achievement). This finding is of particular relevance, as it reveals a conflict between the values underlying cooperative learning and those underlying some of the most common practices in educational environments, such as assessment, certification, competition and rewards.

This research demonstrates that it may well be the overall coherence between the pedagogical methods and values expressed by teachers, on the one hand, and those they perceive to be embedded in their contexts, on the other hand, that is the key factor supporting the implementation of cooperative learning in education.

## Appendix 1

### Study 2, Empirical part 2

In the self-enhancement condition, participants read the following text:
GOOD ECONOMY AMBITIOUS AND POWERFUL ... Our conception of the good economy depends on our model of well-being ... I propose that a career crowned with success and power is at the heart of good living. What counts today is being ambitious, influential and of course successful. Having power over others, authority and being rich can give a positive self-image and social recognition ... In the same way, an economy based on ambition and personal success is really a good economy.

In the self-transcendence condition, participants read the following text: GOOD ECONOMY EQUALITY AND PEACE ... Our conception of the good economy depends on our model of well-being ... I propose that a career dedicated to helping others and protecting the environment is at the heart of good living. What counts nowadays is to be honest, loyal and of course to have an open mind about others. Working for social justice, equality and responsible management makes it possible to become wise and act for the harmony of the world ... In the same way, an economy based on equality and peace is really a good economy In the control condition, participants read the following text: GOOD ECONOMY MODEL OF WELL LIVING ... Our conception of the good economy depends on our model of well-being ... I propose that the model of well-being is based on several factors essential to the well-being of individuals in society. What counts nowadays is to identify these factors, to know them and of course to take them into account. Knowing how to identify these factors, to recognize them makes it possible to live better ... In the same way, an economy based on this model of good living is really a good economy.

## Appendix 2

### Study 4, Empirical part 2

Examples of images to which participants were exposed to by condition.

In the self-enhancement condition: ... our school has 590 students in 27 different classes. They are supervised by a complete teaching team composed of teachers, specialist teachers and teachers in charge of pedagogical support. This institution disposes the necessary material resources for developing ambitious educational projects, in order to attain its goal: a school of excellence able to meet the needs of a world in constant evolution. To fulfil this mission, our school relies on certain fundamental values. We recognize that personal success, ambition and power are essential in our school. These are the values ​​that underlie the implementation of our program, and contribute to maintain a competent community, to preserve a positive image and to ensure the recognition and success of deserving pupils. It is on these bases that our school founded, and that it manages to educate ambitious citizens prepared for personal challenges ...

In the self-transcendence condition: ... our school has 590 students in 27 different classes. They are supervised by a complete teaching team composed of teachers, specialist teachers and teachers in charge of pedagogical support. This institution disposes the necessary human resources for developing educational projects based on the mutual aid between the various actors, in order to attain its goal: a tolerant school ready to meet the needs of a world in constant evolution. To fulfil this mission, our school relies on certain fundamental values. We recognize that equality, mutual respect, and social justice are essential in our school. These are the values ​​that underlie the implementation of our program, and contribute to maintain a caring community, preserve a harmonious and supportive environment, and to ensure the growth of each. It is on these bases that our school is founded, and that it manages to educate honest and loyal citizens ...

In the control condition: ... our school has 590 students in 27 different classes. They are supervised by a complete teaching team composed of teachers, specialist teachers and teachers in charge of pedagogical support. This institution disposes the necessary resources for developing relevant educational projects in order to attain its goal: a school ready to meet the needs of a constantly changing world. To fulfil this mission, our school relies on certain fundamental values. We recognize that the values promoted in this school are essential for the functioning of our institution. These values underlie the implementation of our program and contribute to maintain a community of actors invested in their mission within the school. It is on these bases that our school is organized, and that it manages to educate citizens adapted to life in our society ...

## Footnotes

1 Beliefs and attitudes are two interrelated constructs in cognition that may refer, respectively, to cognitive and affective judgements, (or evaluations) regarding specific entities (Fishbein, 1967).

2 All studies reported in the present paper also included a series of other variables. The reason for including other measures that were not directly related to our research questions was because a) the size of our target population was restrained due to the limited number of individuals who qualify to enrol in the course each year (in the case of students in educational sciences and of pre-service teachers) and b) potential participants were difficult to reach (in the case of in-service teachers).

3 A part of this sample was used in a research project by a student in Educational Sciences (Bourquin, 2011).

4 The distribution of the sample across the four years was as follows: Year 1: 23.48%, Year 2: 22.61%, Year 3: 35.65%, Year 4: 18.26%.

5 This measure comprised 34 items of the 40 items in the original scale. Moreover, instead of asking participants to compare themselves to another person, to make it more comprehensive for participants, each item was formulated to be directly addressed to the respondent (e.g., “It is important to him” in the original scale became “It is important for me” in the adapted scale).

6 All statistical analyses performed using Stata 15 (StataCorp., 2017).

7 No difference across the different years was observed.

8 Participants were asked with an open-ended question to report the educational program in which they were enrolled. However, the majority of the sample reported only the faculty without further specifying the level and the orientation of their studies.

9 Two participants were removed from the sample: the first gave the same answer to all items on the questionnaire and the second had an important number of missing values.

10 For example, the following item: “I wish that my pupils would share their ideas and the means they dispose” was changed to “It is desirable for pupils to share their ideas and the means they dispose”.

11 We note that results from the OLS model with robust standard errors were similar and yielded identical effect sizes: ST values (*b* = .028, *SE* = .009, t(75) = 3.29, *p* = .002) Contrast 1 (*b* = -.009, *SE* = .007, t(75) = -1.31, *p* = .193), contrast 2, (*b* = .015,* SE* = .012, t(75) = 1.26, *p* = .213), contrast 1*ST values (*b *= -.009, *SE* = .006, t(75) = -1.45, *p* = .152, contrast 2*ST values,(*b* = 006, *SE *= .01, t(75) = .59, p = .556.

12 For exploration purposes and for potential utility for future research, we ran a power analysis to estimate the average sample size we would ideally need to achieve 80% power. Analysis showed that, on average, 250–300 observations would be necessary for a small–medium effect size.

13 An item corresponding to conservative values with insignificant loading was removed to improve the measurement validity of the model and its reliability as measured by Cronbach’s *α*.

14 A fixed effect instead of random effect meta-analysis was preferred because the two following conditions were satisfied: a) the design was identical among the different studies and b) the goal of the analysis was to estimate the effect size for a specific population and not to generalize to other populations.

15 For Studies 2, 3, 4 we used the effects of the interactions contrast 1*personal adherence to ST values. For Study 5 we used the effects of the interaction: School adherence to SE*personal adherence to ST relative adherence to ST values.

16 Z-test examines whether the score of the pooled ES is different from zero.
